# Correlation between different levels of thyroid autoantibodies and immune checkpoint inhibitor-associated thyroid dysfunction

**DOI:** 10.3389/fendo.2025.1620718

**Published:** 2025-08-13

**Authors:** Yanyan Jiao, Meihua Xu, Xiaopang Rao

**Affiliations:** ^1^ Department of Nursing, People’s Hospital of Chengyang District, Qingdao, Shandong, China; ^2^ Department of Endocrinology, People’s Hospital of Chengyang District, Qingdao, Shandong, China

**Keywords:** neoplasms, thyroid gland, immune checkpoint inhibitors, immune-related adverse events, anti-thyroid peroxidase antibody, thyrotropin receptor antibody, anti-thyroglobulin antibody

## Abstract

**Background:**

To investigate the correlation between thyroid immune-related adverse events (irAEs) and thyroid autoantibodies in cancer patients treated with immune checkpoint inhibitors (ICIs).

**Methods:**

A retrospective analysis was conducted on 316 cancer patients (139 females, 177 males; median age 64.0 [56.0–71.0] years) treated at Qingdao Chengyang District People’s Hospital from January 2018 to December 2023. Patients were divided into a euthyroid group (*n* = 158) and a thyroid irAEs group (*n* = 158) based on the occurrence of thyroid dysfunction post-ICI therapy. The researchers received at least one treatment with ICIs, and after the initial treatment, they underwent at least one or more tests for thyroid hormone levels, TPOAb, TRAb, and TgAb, with an interval of 4 weeks or more for each test. Thyroid hormone levels and autoantibodies (TPOAb, TRAb, TgAb) were measured. Clinical characteristics and baseline thyroid autoantibodies were evaluated for their association with thyroid irAEs.

**Results:**

Thyroid irAEs included subclinical thyrotoxicosis (19.94%, *n* = 63), clinical thyrotoxicosis (2.53%, *n* = 8), subclinical hypothyroidism (6.01%, *n* = 19), and clinical hypothyroidism (21.52%, *n* = 68). Baseline thyroid autoantibodies were positive in 28.48% (*n* = 45) of the irAEs group versus 5.70% (n = 9) in the euthyroid group (*P* < 0.001). Post-ICI treatment, the thyrotoxicosis group exhibited higher TRAb titers but lower TPOAb titers and TSH levels compared to the hypothyroidism group (*P* < 0.05). Logistic regression identified pre-treatment TRAb positivity (*OR*=6.927, 95% *CI*: 1.817–32.724, *P*=0.002) and TPOAb positivity (*OR* = 7.128, 95% *CI*: 1.877–37.225, *P* = 0.001) as risk factors for thyroid irAEs.

**Conclusion:**

Patients with malignant tumors who had high levels of TPOAb and/or TRAb before treatment were more likely to develop thyroid immune-related adverse events (irAEs) after treatment. The importance of screening for baseline thyroid autoantibodies in predicting thyroid irAEs needs to be clearly understood, and close monitoring and notification to patients should be carried out, along with prior intervention.

## 1 Introduction

In recent years, cancer immunotherapy, particularly immune checkpoint inhibitors (ICIs), has emerged as a breakthrough treatment for malignancies. ICIs target cytotoxic T-lymphocyte-associated antigen-4 (CTLA-4) or programmed cell death-1 (PD-1)/programmed cell death-ligand 1 (PD-L1) pathways to disrupt tumor immune evasion (1). While ICIs are generally well-tolerated, they can induce immune-related adverse events (irAEs) affecting the gastrointestinal tract, endocrine system, skin, and other organs (2). Endocrine irAEs frequently involve thyroid dysfunction (including hypothyroidism with or without transient thyrotoxicosis), occurring in 40% of patients (3, 4). Combination PD-1/CTLA-4 inhibitors pose the highest risk, followed by PD-1 inhibitors alone (4, 5).

Thyroid autoantibodies—anti-thyroid peroxidase antibody (TPOAb), thyrotropin receptor antibody (TRAb), and anti-thyroglobulin antibody (TgAb)—have shown inconsistent associations with thyroid irAEs. Some studies suggest TPOAb/TgAb positivity predicts ICI-related thyroid dysfunction (6–9), while others report no correlation (10). Notably, TRAb’s role remains unexplored (11). To understand whether the occurrence of thyroid irAEs in tumor patients after ICI treatment is related to the patients’ baseline thyroid antibodies, this study investigates the relationship between thyroid autoantibodies and irAEs post-ICI therapy, providing a theoretical basis for clinical intervention. In order to understand whether the occurrence of thyroid immune-related adverse events (irAEs) in tumor patients after using immune checkpoint inhibitors (ICIs) is related to their baseline thyroid antibodies, this study explored the correlation between the occurrence of thyroid irAEs in tumor patients after using ICIs and the levels of thyroid autoantibodies, in order to provide a theoretical basis for intervention treatment.

## 2 Materials and methods

### 2.1 Study population

Patients (*n* = 316) treated with ICIs (Tislelizumab, Pembroliumab, and Xindiliumab) at Qingdao Chengyang District People’s Hospital (2018–2023) were retrospectively analyzed. Based on thyroid irAEs post-ICI, they were stratified into euthyroid and irAEs groups, with the latter further subclassified into thyrotoxicosis (subclinical/clinical) and hypothyroidism (subclinical/clinical). The required sample size was estimated using Kendall’s rule (10 times the variable count). With 20 variables, the baseline sample size was 200; after a 10% buffer for invalid responses, the final target was ≥ 220 cases. Therefore, the inclusion of 316 participants was sufficient for the sample size. Inclusion criteria: The investigator received at least 1 ICIs treatment, and at least 2 or more thyroid hormone levels and TPOAb, TRAb and TgAb tests after initial treatment, with an interval of more than 2 weeks between each test. Exclusion criteria: Prior ICI use, pre-existing thyroid dysfunction, central hypothyroidism, incomplete data, or confounding factors (e.g., tyrosine kinase inhibitors, non-thyroidal illness syndrome, radiation thyroiditis). Ethical approval was obtained (No. 2023100812); informed consent was waived for retrospective data.

### 2.2 Methods

#### 2.2.1 Data collection

Demographics, ICI type, tumor type, and thyroid function tests, including free thyroxine (FT4), reference range 12.0-22.0pmol/l, free triiodothyronine (FT3), reference range 3.5-6.8pmol/l, thyroid-stimulating hormone (TSH), reference range 0.4-4.2uIU/ml, Thyroid stimulating hormone receptor antibody (TRAb), reference range0-1.75IU/L, anti-thyroid peroxidase antibody (TPOAb), reference range 0–34 IU/ml, anti-thyroglobulin antibody (TgAb), reference range 0–115 IU/ml, were recorded. Thyroid function was monitored every 4 weeks (every 2 weeks if abnormal).

#### 2.2.2 Diagnostic criteria

To minimize selection bias, case allocation followed these three principles (12) (1): At least 50 patient cases were collected annually (2); Cases were evenly distributed across each quarter of the year (3); Monthly case contributions accounted for no less than 10% of the total. Patient selection flowchart: Eligible cancer patients were those who received immune checkpoint inhibitor (ICI) therapy. Methods for Assessing Thyroid Function and Severity of Thyroid Injury: According to relevant guidelines for the diagnosis and treatment of thyroid diseases, thyroid injury is classified into the following five categories (1): Thyrotoxicosis: Elevated FT3 (normal reference range: 3.5–6.8 pmol/L) and/or elevated FT4 (normal reference range: 12.0–22.0 pmol/L), with decreased TSH (normal reference range: 0.4–4.2 μIU/mL) (2). Hypothyroidism: FT3 and/or FT4 below the normal reference range, with TSH above the normal reference range (3). Subclinical thyrotoxicosis or subclinical hypothyroidism**: FT3 and FT4 within the normal reference range, with TSH below or above the normal reference range (4). Antibody-positive euthyroidism: Normal TSH, FT3, and FT4, with positive TPOAb (normal range: 0–34 IU/mL) and/or positive TgAb (normal range: 0–115 IU/mL) and/or positive TRAb (normal range: 0–1.75 IU/L) (5). Normal thyroid function: All parameters (TSH, FT3, FT4, and thyroid antibodies) within their respective normal reference ranges (13). The inclusion criteria required: Baseline thyroid function tests, including antibody assessment; At least one post-treatment thyroid function evaluation; Complete collection of essential demographic and clinical data. Thyrotoxicosis: Subclinical: TSH < lower limit of normal (LLN), normal FT4/FT3. Clinical: TSH < LLN, elevated FT4/FT3. Hypothyroidism: Subclinical: TSH > upper limit of normal (ULN), normal FT4. Clinical: TSH > ULN, FT4 < LLN.

### 2.3 Statistical analysis

Statistical analyses were performed using SPSS 27.0 (IBM Corp.). Continuous variables were assessed for normality using Shapiro-Wilk tests. Normally distributed data were presented as mean ± standard deviation (*SD*) and compared using Student’s t-test, while non-normally distributed data were expressed as median (interquartile range, Q1-Q3) and analyzed with Mann-Whitney U test. Categorical variables were compared using *χ* ^2^ test or Fisher’s exact test, as appropriate. To address potential type I error inflation in multiple comparisons, we applied Bonferroni correction when appropriate, with adjusted *P*-values reported for primary outcomes. Binary logistic regression analysis was conducted to identify independent risk factors for immune-related adverse events (irAEs), incorporating clinically relevant covariates and variables showing *P* < 0.10 in univariate analyses. Model assumptions were verified including absence of multicollinearity (variance inflation factors < 5) and adequate goodness-of-fit (Hosmer-Lemeshow test *P* > 0.05). A two-tailed *P*-value < 0.05 was considered statistically significant, except where multiple comparison adjustments were applied.

## 3 Results

### 3.1 Clinical characteristics

**Table 1** presented a comparative analysis of baseline characteristics between the euthyroid group (*n* = 158) and the thyroid irAEs group (*n* = 158) in 316 cancer patients treated with immune checkpoint inhibitors (ICIs). No significant differences were observed in sex distribution (47.48% vs. 49.37% male, *P* = 0.282) or median age (66.0 vs. 62.5 years, *P* = 0.095) between groups. The majority received PD-1/PD-L1 inhibitors (100% in euthyroid vs. 92.41% in irAEs group), with combination CTLA-4/PD-1/PD-L1 therapy exclusively administered to the irAEs group (7.59%, *P* = 0.513 by Fisher’s exact test). Tumor types differed significantly (*P* = 0.032), with lung cancer predominating in the euthyroid group (78.48% vs. 58.23%) and gynecologic cancers more frequent in the irAEs group (12.02% vs. 1.90%).

**Table 1:** Comparison of general information between the normal thyroid function group and the thyroid irAEs group.

Variables	Total (*n* = 316)	Normal thyroid function group (*n* = 158)	Thyroid irAES group (*n* = 158)	*χ²*/*Z* value	*P* value
Male [*n* (%)]	153 (48.42)	75 (47.48)	78 (49.37)	1.12	0.282
Age [years *M* (Q1, Q3)]	64.0 (56.0, 71.0)	66.0 (59.0, 72.3)	62.5 (56.0, 71.3)	-1.41	0.095
ICI type [*n* (%)]				–	0.513
PD-1/PD-L1 inhibitors	304 (96.20)	158 (100.0)	146 (92.41)		
CTLA-4 and PD-1/PD-L1 inhibitors	12 (3.80)	0 (0)	12 (7.59)		
Tumor type [*n* (%)]				–	0.032
Lung cancer	216 (68.35)	124 (78.48)	92 (58.23)		
Gastrointestinal cancer	48 (15.19)	20 (12.66)	28 (17.72)		
Gynecologic cancer	22 (6.96)	3 (1.90)	19 (12.02)		
Urologic cancer	27 (8.54)	11 (6.96)	16 (10.13)		
Non-Hodgkin lymphoma	3 (0.95)	0 (0)	3 (1.90)		
TSH [mU/L, *M* (Q1, Q3)]	1.59 (0.94, 2.43)	1.92 (1.22, 2.94)	1.40 (0.92, 2.30)	-3.16	0.023
Thyroid antibody positivity [*n* (%)]	54 (17.09)	9 (5.70)	45 (28.48)	12.18	< 0.001
TgAb +	9 (2.85)	3 (1.90)	6 (3.80)		
TPOAb +	14 (4.43)	3 (1.90)	11 (6.96)		
TRAb +	2 (0.63)	0	2 (1.26)		
TPOAb-TRAb +	1 (0.32)	0	1 (0.64)		
TgAb-TRAb +	0	0	0		
TPOAb-TgAb +	26 (8.23)	3 (1.90)	23 (14.56)		
TPOAb-TgAb-TRAb +	2 (0.63)	0	2 (1.26)		
Thyrotoxicosis [*n* (%)]	71 (22.46)	–	71 (44.93)	–	–
Clinical thyrotoxicosis	8 (2.53)	–	8 (5.06)	–	–
Subclinical thyrotoxicosis	63 (19.93)	–	63 (39.87)	–	–
Hypothyroidism [*n* (%)]	87 (27.53)	–	87 (55.06)	–	–
Clinical hypothyroidism	68 (21.52)	–	68 (43.04)	–	–
Subclinical hypothyroidism	19 (6.01)	–	19 (12.02)	–	–

-: Indicates no result.

Key thyroid-related parameters revealed lower baseline TSH levels in the irAEs group (median 1.40 vs. 1.92 mU/L, *P* = 0.023) and markedly higher thyroid autoantibody positivity (28.48% vs. 5.70%, P<0.001). Notably, combined TPOAb-TgAb positivity was prevalent in the irAEs group (14.56% vs. 1.90%). Among irAEs subtypes, hypothyroidism (55.06%) was more common than thyrotoxicosis (44.93%), with clinical hypothyroidism constituting 43.04% of cases.

### 3.2 Autoantibody profiles

**Table 2** compared thyroid hormone profiles and autoantibody levels between the thyrotoxicosis (*n* = 71) and hypothyroidism (*n* = 87) subgroups of patients who developed thyroid immune-related adverse events (irAEs) following immune checkpoint inhibitor (ICI) therapy. The data reveal distinct immunological patterns between these two manifestations of thyroid dysfunction.

**Table 2:** Comparison of thyroid indicators between two groups.

Groups	TPOAb	TgAb	TRAb	FT3	FT4	TSH
Thyrotoxicosis(*n*=71)	11.78 ± 4.72	56.23 ± 25.05	2.56 ± 0.46	4.25 ± 0.88	15.32 ± 4.71	0.11 ± 0.02
Hypothyroidism(*n*=87)	32.14 ± 7.97	54.57 ± 26.48	0.24 ± 0.12	4.15 ± 1.06	15.98 ± 4.08	4.21 ± 1.26
*T*	6.72	0.205	7.934	0.077	1.064	15.206
*P*	0.035	0.815	0.019	0.925	0.588	<0.001

Patients with thyrotoxicosis demonstrated significantly lower thyroid peroxidase antibody (TPOAb) levels (11.78 ± 4.72 IU/mL vs 32.14 ± 7.97 IU/mL, *P* = 0.035) but markedly higher thyrotropin receptor antibody (TRAb) titers (2.56 ± 0.46 IU/L vs 0.24 ± 0.12 IU/L, *P* = 0.019) compared to the hypothyroidism group. These findings suggest different underlying pathophysiological mechanisms, with TRAb potentially driving the thyrotoxicosis phenotype while TPOAb predominates in hypothyroidism. Notably, thyroglobulin antibody (TgAb) levels showed no significant intergroup difference (56.23 ± 25.05 IU/mL vs 54.57 ± 26.48 IU/mL, *P* = 0.815).

As expected, thyroid-stimulating hormone (TSH) levels were profoundly suppressed in thyrotoxicosis (0.11 ± 0.02 mIU/L) versus elevated in hypothyroidism (4.21 ± 1.26 mIU/L, *P* < 0.001). Free thyroid hormones (FT3 and FT4) showed no statistically significant differences between groups, though the numerical values align with the clinical definitions of these conditions.

These results highlighted the importance of differentiating between thyrotoxicosis and hypothyroidism in ICI-related thyroid irAEs, as they exhibit distinct autoantibody profiles that may inform both pathogenesis and clinical management.

### 3.3 Risk factors for thyroid irAEs

**Table 3** presented the logistic regression analysis of risk factors for developing thyroid immune-related adverse events (irAEs) following immune checkpoint inhibitor therapy. The analysis identified pre-treatment thyroid autoantibodies as the most significant predictors of thyroid dysfunction, while demographic and other clinical factors showed no significant association.

**Table 3:** Analysis of immune-related adverse reactions risk factors for thyroid irAEs group.

Indicators	*β* value	*SE* value	*Wald χ^2^* value	*OR* value (95% *CI*)	*P* value
Gender	0.234	0.411	0.333	0.812 (0.457-1.293)	0.612
Age	-0.312	0.125	0.738	0.667 (0.417-1.461)	0.318
TSH	-0.178	0.315	0.499	0.582 (0.264-1.217)	0.471
TgAb	0.504	0.262	0.181	0.684 (0.321-1.524)	0.503
TPOAb	2.016	0.879	7.128	8.304 (1.877-37.225)	0.001
TRAb	1.890	0.732	6.927	6.984 (1.817-32.724)	0.002

Notably, thyroid peroxidase antibody (TPOAb) positivity demonstrated the strongest association with thyroid irAEs (*OR*=8.30, 95% *CI*: 1.88-37.23, *P* = 0.001), followed by thyrotropin receptor antibody (TRAb) positivity (*OR*=6.98, 95% *CI*: 1.82-32.72, *P* = 0.002). These findings suggest that pre-existing thyroid autoimmunity substantially increases susceptibility to ICI-induced thyroid dysfunction. In contrast, thyroglobulin antibody (TgAb) status (*OR* = 0.68, 95% *CI*: 0.32-1.52, *P* = 0.503), age (*OR* = 0.67, 95% *CI*: 0.42-1.46, *P* = 0.318), gender (*OR* = 0.81, 95% *CI*: 0.46-1.29, *P* = 0.612), and baseline TSH levels (*OR* = 0.58, 95% *CI*: 0.26-1.22, *P* = 0.471) were not statistically significant risk factors in this cohort.

These results emphasize the critical importance of pre-treatment thyroid autoantibody screening, particularly for TPOAb and TRAb, as these markers may help identify patients at highest risk for developing thyroid irAEs during ICI therapy. The differential predictive values of specific autoantibodies suggest distinct pathophysiological mechanisms underlying different forms of ICI-related thyroid dysfunction.

## 4 Discussion

In recent years, immune checkpoint inhibitors (ICIs) have been widely adopted in the treatment of various malignancies, significantly improving patient prognosis. However, while enhancing antitumor immunity, these agents can induce excessive immune responses in normal tissues, leading to immune-related adverse events (irAEs) (14). Endocrine organs—particularly the pituitary, thyroid, and pancreas—are frequent targets of such irAEs. The role of baseline immune status in predisposing cancer patients to thyroid irAEs remains controversial.

Several studies have demonstrated that patients with pre-existing thyroid autoantibodies (e.g., TPOAb, TRAb, TgAb) are more susceptible to thyroid dysfunction following PD-1 inhibitor monotherapy or combination PD-1/CTLA-4 blockade (15–17). In contrast, other reports suggest that thyroid irAE development correlates poorly with autoantibody titers (10, 18), implying a more complex interplay between multiple immune pathways (16, 19). Our findings align with the former perspective, as baseline thyroid autoantibody positivity was significantly higher in the irAEs group than in euthyroid controls (28.48% vs. 5.70%, P<0.001). This supports the hypothesis that pre-treatment autoimmunity primes the thyroid for ICI-mediated injury. TgAb was produced after thyroglobulin enters the bloodstream and can bind with thyroglobulin to form complexes, which have a destructive effect on thyroid follicular epithelial cells. Unlike TPOAb, thyroglobulin is not expressed on the surface of thyroid cells, limiting the antibody-dependent cell-mediated cytotoxicity of TgAb. A positive result is only observed in 60% of patients with autoimmune thyroid diseases (20).

The prognostic implications of specific autoantibodies remain unclear. Prior research indicates that while both TPOAb and TgAb predict thyroid irAEs, their clinical trajectories differ: TgAb positivity often precedes thyrotoxicosis, whereas TPOAb-positive patients tend to progress from transient thyrotoxicosis to persistent hypothyroidism (21). Our data further refine this model by revealing significantly higher TRAb titers in thyrotoxicosis cases versus hypothyroidism (*P* < 0.05), alongside lower TPOAb/TSH levels—a pattern suggesting TRAb’s potential role in Graves’-like thyrotoxicosis. Notably, TgAb levels showed no difference for the two groups, contradicting earlier reports that identified TgAb as a risk factor for PD-1 inhibitor-induced thyroiditis (8). Sex disparities in irAE susceptibility remain unclear; while Muir et al. (22) reported higher predisposition in females, our analysis—consistent with reference (8)—found no sex-based association.

The pathophysiology of ICI-induced thyroiditis likely involves both cellular and humoral immunity. Murine models demonstrate CD4+ T-cell infiltration in thyroid tissue during irAEs, implicating Th1/Th17-driven inflammation in destructive thyroiditis (23). Human studies similarly link thyroid peroxidase (TPO)- and thyroglobulin (Tg)-specific CD8+ T cells to Hashimoto’s-like damage (24). Intriguingly, Tg-directed immunity may dominate in PD-1 inhibitor-associated cases (8), as evidenced by marked TgAb/TPOAb elevation post-treatment in a hypothyroidism patient (25). These observations collectively underscore thyroid irAEs as a multifaceted immune phenomenon warranting mechanistic exploration.

Several limitations should be acknowledged in this study. Firstly, the single-center, retrospective design may limit generalizability and introduce selection bias. Second, as a cross-sectional analysis, we could not establish causal relationships between thyroid autoantibodies (TPOAb/TRAb) and thyroid dysfunction outcomes following ICI therapy - the observed associations require validation through prospective intervention studies. Third, potential confounding factors including concurrent medications and pre-existing autoimmune conditions may influence results. This study identifies pre-treatment TPOAb/TRAb as practical biomarkers for thyroid irAE risk stratification, advocating their routine screening before ICI initiation. However, as a single-center retrospective analysis, this work cannot establish causality or delineate autoantibody-specific risks for thyrotoxicosis versus hypothyroidism. Prospective multicenter studies with serial immune profiling are needed to validate these findings and guide personalized monitoring protocols.

## Data Availability

The raw data supporting the conclusions of this article will be made available by the authors, without undue reservation.
